# Genetic diversity of historical Atlantic walruses (*Odobenus rosmarus rosmarus*) from Bjørnøya and Håøya (Tusenøyane), Svalbard, Norway

**DOI:** 10.1186/s13104-016-1907-8

**Published:** 2016-02-18

**Authors:** Charlotte Lindqvist, Tilottama Roy, Christian Lydersen, Kit M. Kovacs, Jon Aars, Øystein Wiig, Lutz Bachmann

**Affiliations:** Department of Biological Sciences, University at Buffalo (SUNY), Buffalo, NY 14260 USA; Department of Ecology and Evolutionary Biology, University of Michigan, Ann Arbor, MI 48109 USA; Norwegian Polar Institute, Fram Centre, N-9296 Tromsø, Norway; Natural History Museum, University of Oslo, PO Box 1172, Blindern, 0318 Oslo, Norway

**Keywords:** Ancient DNA, Genetic bottleneck, Mitochondrial DNA, Over-exploitation, Sequence diversity, Spitsbergen

## Abstract

**Background:**

The population size of Atlantic walruses (*Odobenus rosmarus rosmarus*) is depleted relative to historical abundance levels. In Svalbard, centuries of over-exploitation brought the walrus herds to the verge of extinction, and such bottlenecks may have caused loss of genetic variation. To address this for Svalbard walruses, mitochondrial haplotypes of historical walruses from two major haul-out sites, Bjørnøya and Håøya, within the Archipelago were explored using bone samples from animals killed during the peak period of harvesting.

**Results:**

Using ancient DNA methodologies, the mitochondrial NADH dehydrogenase 1 (ND1) gene, the cytochrome c oxidase 1 (COI) gene, and the control region (CR) were targeted for 15 specimens from Bjørnøya (of which five were entirely negative) and 9 specimens from Håøya (of which one was entirely negative). While ND1 and COI sequences were obtained for only a few samples, the CR delivered the most comprehensive data set, and the average genetic distance among historic Svalbard samples was 0.0028 (SD = 0.0023).

**Conclusions:**

The CR sequences from the historical samples appear to be nested among contemporary Atlantic walruses, and no distinct mitochondrial haplogroups were identified in the historical samples that may have been lost during the periods of extensive hunting. However, given the low sample size and poor phylogenetic resolution it cannot be excluded that such haplogroups existed.

**Electronic supplementary material:**

The online version of this article (doi:10.1186/s13104-016-1907-8) contains supplementary material, which is available to authorized users.

## Findings

The walrus (*Odobenus rosmarus*) has a circumpolar Arctic distribution. The Atlantic walrus (*O. r. rosmarus*) is distributed from the Canadian Arctic in the west to the Kara Sea in Russia in the east [1, 2]. The population size of Atlantic walruses is depleted relative to historical abundance levels due to over-harvesting. For example, the number of walruses in Central West Greenland (a population shared with Canada) was reduced by about 80 % between 1900 and 1960 [1, 3]. The exact number of total extant Atlantic walruses is not known but is estimated to be close to 35,000 [4]. In Svalbard the first recorded walrus hunt took place in 1604 [5], but the extensive commercial hunting began around 1820 [6]. By the middle of the 19th century the stock showed clear signs of declining, but harvesting continued. The centuries of walrus hunting in the Svalbard Archipelago brought the reportedly large herds to the verge of extinction [7]. They were finally given total protection in 1952. It was estimated that around 1980 there were only approximately 100 individuals summering in Svalbard, most of them males [8]. The population size of North Atlantic walruses in Svalbard has been increasing in recent decades. Lydersen et al. [9] estimated the number of walruses in Svalbard in 2006 to be about 2600. These authors assumed that the total population size including the individuals from Franz Josef Land to be approximately 5000. A more recent estimate from 2012 for Svalbard indicated a continued upward trend in the population size in recent years to approximately 3900 [10].

**Fig. 1:**
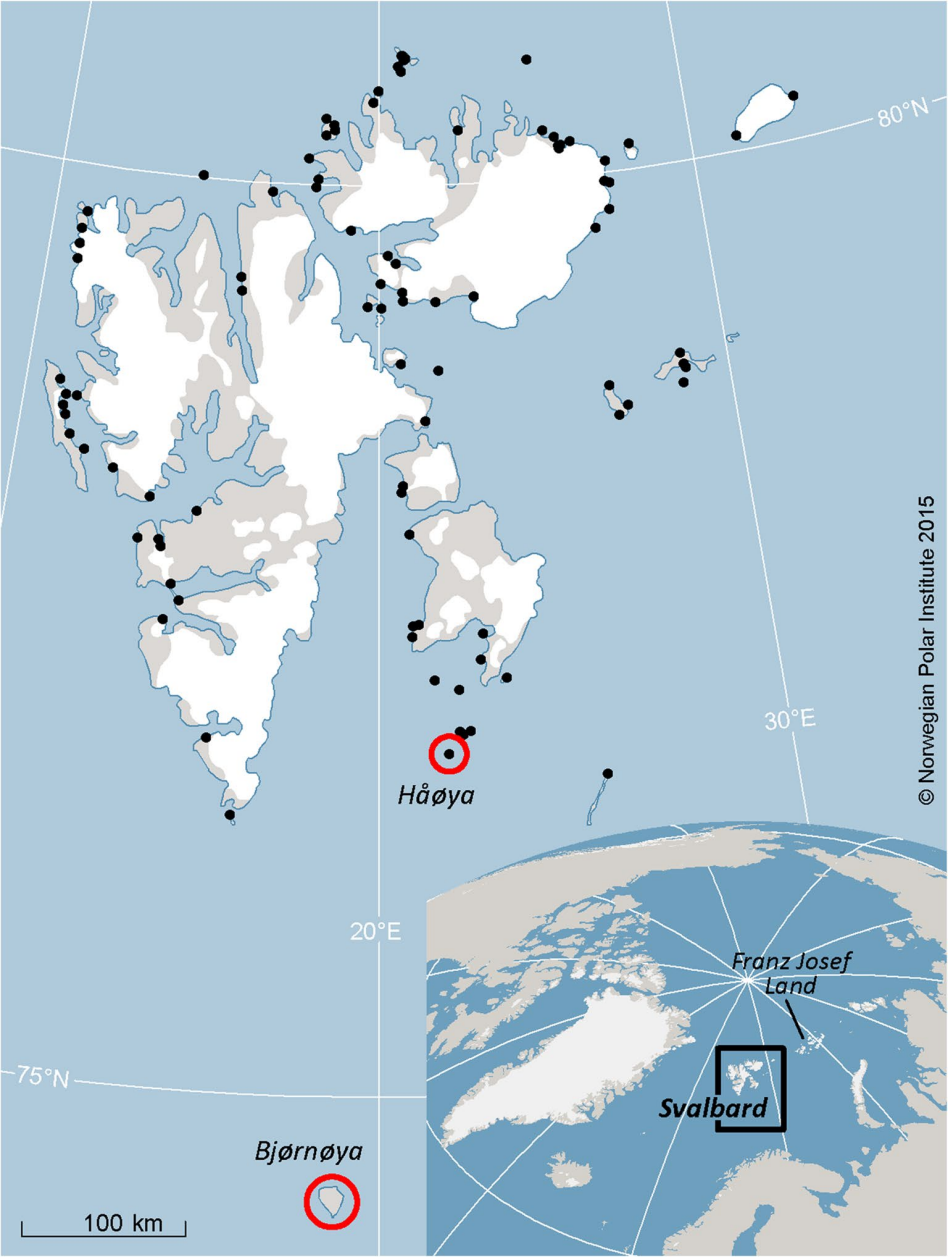
Haul-out sites (*black spots*) of the Atlantic walrus (*O. r. rosmarus*) in the Svalbard Archipelago. Sites where historical samples were collected for this study are encircled in *red*

There is no data on the original walrus population size in Svalbard prior to hunting, but it is thought to have been very large [11, 12]. Substantial declines in population size (bottlenecks) can be accompanied by a loss of genetic variation, and this may have been the case for the walrus populations in the northern Barents Sea. Historically there were four major haul-out regions used by walruses in Svalbard (Fig. 1): (1) Bjørnøya; (2) Southeast Edgeøya including Tusenøyane; (3) Kvitøya and Nordaustland in the northeast; and (4) West- and Northwest Spitsbergen from Isfjorden to Wijdefjorden [13]. In recent decades, walruses have started occupying former haul-out areas throughout the Archipelago (details of these observations can be found in a metadatabase at http://www.npolar.no/en/services/mms/), though sightings on Bjørnøya are still rare [10].

Various studies on the distribution [8, 13], and movement patterns [14–16], as well as population structure [17–19] have shown that the walruses in Svalbard and Franz Josef Land belong to the same population. In the summer months most males stay in Svalbard and most females and calves remain in the northeastern parts of Svalbard and in Franz Josef Land. Migration between the Svalbard–Franz Josef Land population and the neighboring population in East Greenland is thought to be rare. The relationship between the Svalbard–Franz Josef Land population and walruses in Novaya Zemlya and the Pechora and Kara Seas in the southeastern Barents Sea remains unknown [1, 16, 20, 21].

In this study, mitochondrial haplotype diversity of Atlantic walrus in the Svalbard area at the peak of the harvesting period was explored. Bone samples collected from historical haul-out sites on Bjørnøya (15 specimens) and Håøya (nine specimens) were included. Details regarding the samples are presented in Table 1. Unfortunately, radiocarbon dates are not available for these samples. However, the samples from Bjørnøya most likely originated from animals hunted before 1867 because hunting did not occur in this area subsequent to this date [6]. The mandibles collected at Håøya in the Tusenøyane Archipelago are probably remains from one of the most famous slaughters of walruses in Svalbard [22], which occurred in 1852. The exact number of walruses present on the island at that time is not known, but it is believed that several thousands might have been hauled out, and many hundreds were killed in just 1 day [23]. Later, Håøya was only rarely used as a haul-out site by walruses [23, 24], even into the first half of the 20th century [11], though haul-out at this site is more common today [10]. It is therefore likely that the mandibles from Håøya are remains of the 1852 slaughter.

**Table 1:** Historical walrus bone samples analyzed in this study

Sample	Locality	Target region I CR (785 bp)	Target region II ND1 (1966 bp)	Target region III COI (703 bp)	GenBank accession no. target I	GenBank accession no. target II	GenBank accession no. target III
SBj01	Bjørnøya	1–4	8–10, 12	Failed	KU710183	KU710201	n.a.
SBj02	Bjørnøya	1–4	1–12	Failed	KU710184	KU710202	n.a.
SBj03	Bjørnøya	1–4	1–12	Failed	KU710185	KU710203	n.a.
SBj04	Bjørnøya	1–4	2–12	Failed	KU710186	KU710204	n.a.
SBj05	Bjørnøya	Failed	Failed	Failed	n.a.	n.a.	n.a.
SBj06	Bjørnøya	1–4	7–8	Failed	KU710187	KU710205	n.a.
SBj07	Bjørnøya	4	Failed	Failed	KU710188	n.a.	n.a.
SBj08	Bjørnøya	1–4	2–12	Failed	KU710189	KU710206	n.a.
SBj09	Bjørnøya	1–4	2, 7–8	Failed	KU710190	KU710215, KU710216	n.a.
SBj10	Bjørnøya	Failed	Failed	Failed	n.a.	n.a.	n.a.
SBj11	Bjørnøya	1–4	2–3, 5–12	Failed	KU710191	KU710207	n.a.
SBj12	Bjørnøya	1–3	2–3, 5–12	Failed	KU710192	KU710208	n.a.
SBj13	Bjørnøya	Failed	Failed	Failed	n.a.	n.a.	n.a.
SBj14	Bjørnøya	Failed	Failed	Failed	n.a.	n.a.	n.a.
SBj15	Bjørnøya	Failed	Failed	Failed	n.a.	n.a.	n.a.
SH16	Håøya	1–4	1–12	3	KU710193	KU710209	KU710175
SH17	Håøya	1–4	1–12	3	KU710194	KU710210	KU710176
SH18	Håøya	1–3	1–2, 7–8	3	KU710195	KU710217, KU710218	KU710177
SH19	Håøya	1–4	1–12	3	KU710196	KU710211	KU710178
SH20	Håøya	1–4	1–12	3	KU710197	KU710212	KU710179
SH21	Håøya	1–3	1–2, 7–8	3	KU710198	KU710219, KU710220	KU710180
SH22	Håøya	1–4	1–12	3	KU710199	KU710213	KU710181
SH23	Håøya	Failed	Failed	Failed	n.a.	n.a.	n.a.
SH24	Håøya	1–4	1–4, 6–12	3	KU710200	KU710214	KU710182

The successfully sequenced mitochondrial target regions are indicated (for numbering and details on the targeted regions see Table 2)

*n.a.* not applicable

Ancient DNA methodologies used in this study followed the protocols described previously [25]. In short, DNA was extracted from 0.1–0.5 g of finely ground bone powder as described in [26]. Standard precautions and measures for ancient DNA analyses demonstrating authenticity of the obtained data were followed. The mitochondrial NADH dehydrogenase 1 (ND1) gene was targeted with amplification of 12, the cytochrome c oxidase 1 (COI) gene of four, and the control region (CR) of four overlapping fragments (Table 2). Herein, we report the nucleotide sequences obtained and their possible implications for the genetic structure of the Atlantic walruses in the Svalbard Archipelago.

**Table 2:** PCR primers used in this study for the amplification of the mitochondrial NADH dehydrogenase 1 (ND1) and cytochrome c oxidase subunit 1 (COI) genes, as well as the control region (CR) from aDNA extracts from bones of Atlantic walrus (*Odobenus rosmarus*) from Svalbard

Amplicon	**Primer**	**Forward primer sequence (5′–3′)**	**Primer**	**Reverse primer sequence (5′–3′)**
ND1-1	ND1-1F^a^	ACCCCGCCTGTTTACCAAAAACAT	ND1-1R	TATATTCCCGCCTCTTCACG
ND1-2	ND1-2F	GCCACACGAGGGTTCTACTG	ND1-2R	TCGGGGGTTGTGCTATACTC
ND1-3	ND1-3F	GGGACAGCAATTTAGGTTGG	ND1-3R^b^	TGTCCTGATCCAACATCGAG
ND1-4	ND1-4F^b^	GGGATAACAGCGCAATCCTA	ND1-4R	TAGTCCTAAGGCGCTTCGTC
ND1-5	ND1-5F	CAAGAGAGACAAGGCCCACT	ND1-5R	GGAAGGCTACGGCTAGAAGAA
ND1-6	ND1-6F	CTTTTACCCCCAGAGGTTCA	ND1-6R^b^	TGGTCGTAAAGGTTCCTTGG
ND1-7	ND1-7F^b^	GGACCATACGGACTTCTCCA	ND1-7R	CGGCTAGGCTTGATATTGCT
ND1-8	ND1-8F	CCTATACCGTACCCTCTCATCA	ND1-8R	TGGTTAAAGAGAATGATCCGTTT
ND1-9	ND1-9F	GCCATCATTCTCTTGTCCGTA	ND1-9R^b^	CCAGGAAGAATAGGGCGAAT
ND1-10	ND1-10F^b^	CAGAATTAGTATCAGGCTTCAACG	ND1-10R	ATGATGCCCGAATTCATAGG
ND1-11	ND1-11F	TTCACAACCACTCTATTTCTTGGA	ND1-11R	TTGTGGAGGAACACTTGCTG
ND1-12	ND1-12F	TGCATATGACACATGGCTCTG	ND1-12R^b^	GGTATGGGCCCGATAGCTTA
COI-1	COI-1F^b^	ACAAGGACATCGGCACTCTC	COI-1R^b^	GCTCCGATTATTAGGGGAACT
COI-2	COI-2F^b^	GCCCATGCATTCGTAATAATC	COI-2R^b^	GATGGTCCTGCGTGAGCTA
COI-3	COI-3F^b^	GAACCGGATGAACCGTCTAC	COI-3R^b^	CCGCTGTAATTAATACGGACCA
COI-4	COI-4F^b^	CTCCCGCAATATCCCAATAC	COI-4R^b^	TTCCGAATCCTGGTAGAATGA
CR-1	CR-1F^b^	GCCTATTGCCGGTATAATCG	DL-1R^b^	TGTGATGGTACAGTAGGGGTGA
CR-2	CR-2F^b^	CTGACGCCCTACCATTCATA	DL-2R^b^	AAGGGTTGCTGGTTTCTCG
CR-3	CR-3F^b^	AATTCACTTGGTCCGTCAAGC	DL-3R^b^	TTATGTGTGATCATGGGCTGA
CR-4	CR-4F^b^	TGGGACATCTCGATGGACTT	DL-4R^b^	CGTGTATGTCCTGTGACCATT

Primers were designed based on sequences of the complete mitochondrial genome (GenBank accession AJ428576; [34]

^a^ [35]

^b^ [25]

Five samples from Bjørnøya (SBj05, SBj10, SBj12, SBj13, and SBj14)  and one sample from Håøya (SH23) did not deliver any PCR products, and it was therefore concluded that no DNA survived in these bones. The SBj07 samples delivered only a short sequence for the mitochondrial control region. PCR success depended on the specific target region suggesting that some primer pairs were not optimally designed for the purpose of amplifying degraded walrus target DNA. This may be particularly the case for some ND1, but also to some extent for the targeted COI and CR fragments (Table 1), although primer pairs for the latter targets were successfully used in earlier studies [25].

### Cytochrome c oxidase 1 (COI)

PCR amplification and sequencing of the COI was only successful for the 177 bp comprising region 3 for eight of the nine Håøya samples (Additional file 1). No sequence information was obtained for any of the 15 samples from Bjørnøya; all attempts for PCR amplification failed for DNA extracts from samples of this site. Two haplotypes were detected for the Håøya samples that differed by only one synonymous transition.

### NADH dehydrogenase 1 (ND1)

Nucleotide sequence information was determined for samples from both Bjørnøya and Håøya (Additional file 2) for this gene. Again, a higher proportion of Håøya samples delivered nucleotide sequences than those from Bjørnøya. Nevertheless, for seven samples (two from Bjørnøya and five from Håøya) full sequence information was obtained. Nucleotide substitutions were detected at only six sites leading to seven distinct haplotypes for these samples.

### Control region (CR)

This target region delivered sequence information for 18 samples (Additional file 3). Nucleotide substitutions were detected at eight positions. In the sequence obtained for SH20 one position remained ambiguous. A variable number of nucleotides were observed in a polyT/polyC region. This could reflect either natural variation or degradation of the old template DNA. However, similar variation was also observed in modern sequences (see respective sequences from available GenBank entries in Additional file 3), which may be taken as an indication that this reflects real natural variation. Nevertheless, if this variable polyT/polyC region is not considered there were seven distinct haplotypes in the old dataset.

**Fig. 2:**
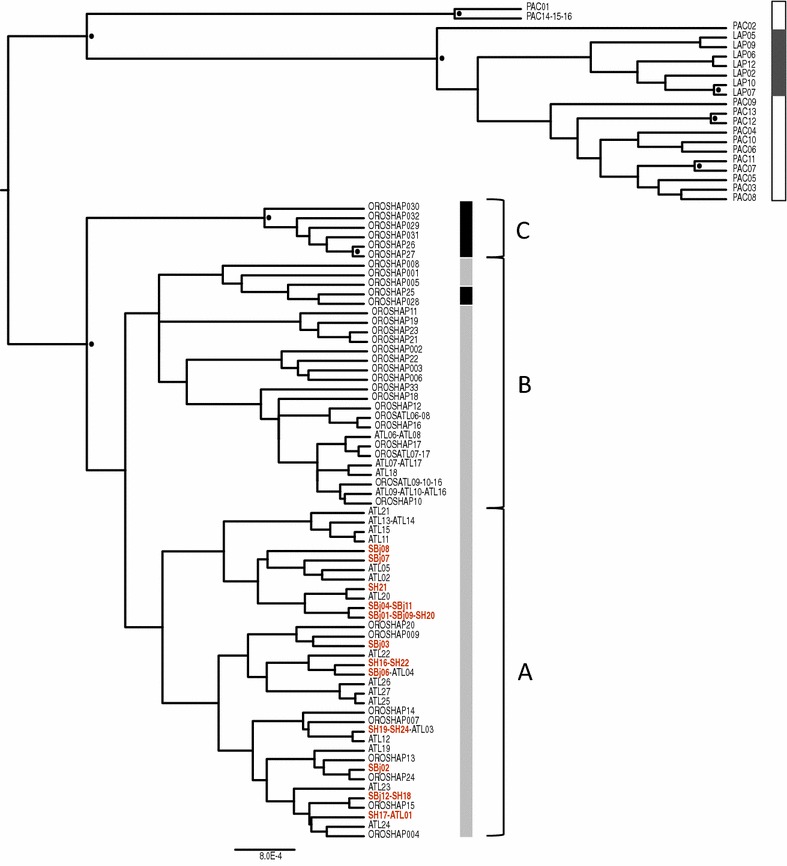
Maximum clade credibility tree reconstructed from a BEAST analysis of mitochondrial control region sequences obtained in this study of historical samples from Bjørnøya and Håøya, and Atlantic and Pacific walrus sequences downloaded from GenBank (Additional file 4: Table S1). Samples of walruses from the Pacific, Laptev, Atlantic, and Maritime regions are shown with *white*, *dark gray*, *light gray*, and *black bars*, respectively, and samples from Bjørnøya and Håøya are shown in *red*.* Black circles* indicate Bayesian posterior probability values of 0.9 and above. Clades *A*, *B*, and *C* that are discussed in the main text are indicated

The obtained CR sequences can be compared to two other recent studies that also reported mitchondrial CR sequence variation for walruses in the North Atlantic region. Lindqvist et al. [25] assessed the taxonomic status of the Laptev walrus (*O. r. laptevi*), including nine samples from the Tusenøyane (Svalbard), five samples from Franz Josef Land, and another five samples from Scoresby Sound (East Greenland). McLeod et al. [27] compared walrus specimens from the eastern Canadian Arctic, including the extirpated southeastern Canadian Maritimes walrus. Available CR sequences from these previous studies were downloaded from GenBank (Additional file 4: Table S1) and compared to the new data generated from the historical Håøya and Bjørnøya samples. The most comprehensive dataset was assembled for the CR, including information from 88 haplotypes (Additional file 3). Since only a few nucleotide sequences were obtained for COI and ND1, they will not be discussed further, and in the following, we focus on the discussion of the results obtained for the CR sequences. The CR dataset was subjected to maximum likelihood and Bayesian phylogenetic analyses in order to address whether the historical samples from Håøya or Bjørnøya could be assigned to specific mitochondrial lineages. The best-fit nucleotide substitution model was determined for the dataset using jModelTest v.1.1 [28]. The HKY + I model was favored and implemented in the phylogenetic analyses. Bayesian analyses were run in BEAST v.1.8 package [29] with the Yule process tree prior, a lognormal relaxed clock model, for 50 million generations. The program Tracer v.1.5 was used to check for stationarity (the ESS was >200 for all the parameters sampled), and TreeAnnotator v.1.8 was used to summarize the set of post burn-in trees and their parameters (burn-in set to 500), and to produce a maximum clade credibility (MCC) phylogeny (shown in Fig. 2). A maximum likelihood tree was reconstructed using the RAxML Blackbox tool [30] available through the Cipres Web Portal (http://www.phylo.org). A phylogenetic network was created with Neighbor-Net [31] in SplitsTree v.4.13.1 [32] using uncorrected p-distances. For genetic distance calculations the most parameterized model available in SplitsTree was chosen, with an HKY85 model, transitions: transversions weighted 2:1, gamma model of rate heterogeneity, with base frequencies estimated empirically. Nucleotide sequence distances for eastern Atlantic walruses (east Greenland, Svalbard, and Franz Josef Land) were calculated using the program DNADIST v.3.5 (copyright J. Felsenstein 1986–2008) as implemented in the program BioEdit v.7.2.5 [33].

The sequences obtained from the Håøya and Bjørnøya samples were very similar to sequences obtained from modern Atlantic walruses from the Svalbard Archipelago. In the Bayesian maximum clade credibility tree, the haplotypes of the Håøya and Bjørnøya samples group within a statistically well supported clade of Atlantic walruses. Within the Atlantic walrus clade there are essentially three subclades (A, B, and C), of which only clade C is statistically supported. The historical Håøya and Bjørnøya samples group within clade A, which also comprises walruses from East Greenland, Svalbard, and Franz Josef Land (Fig. 2). Clades B and C are comprised of modern walruses from West Greenland and Canada, as well as extirpated southeastern Canadian Maritimes walrus. A Maximum Likelihood (ML) analysis (Additional file 5: Figure S1) and a ML analysis on a trimmed dataset (Additional file 6: Figure S2), which was conducted in order to reduce the potential impact of missing information on the tree topology, supported the split between Atlantic and Pacific walruses. Within the Atlantic walrus only clade C received moderate bootstrap support in the analysis with the full dataset. Subclades A and B were rendered unresolved. The median-joining network also recognized the Pacific–Atlantic walrus split, and grouped the haplotypes from the historical samples from Bjørnøya and Håøya with modern walruses from East Greenland, Svalbard, and Franz Josef Land (Additional file 7: Figure S3). It is worth noting that for the most part the Håøya and Bjørnøya samples exhibited distinct haplotypes from modern walruses. Few shared haplotypes were observed between three Håøya individuals (SH17, SH19, SH24) and modern walruses from Tusenøyane in Svalbard (ATL01 and ATL03), and between one Bjørnøya sample and a modern individual from Lågøya in Svalbard (ATL04). Håøya is part of Tusenøyane, whereas Lågøya is situated in the northwest of the archipelago (in contrast to Bjørnøya which is the southernmost island in the archipelago). Based on nucleotide sequence distances between the historical walruses in this study (12 haplotypes), modern walruses from East Greenland, Svalbard, and Franz Josef Land (14 haplotypes), and modern Svalbard walruses (9 haplotypes), the average distances are slightly lower for the historical walruses (0.0028, SD = 0.0023) than for modern eastern Atlantic walruses (0.0050; SD = 0.0024) and Svalbard walruses (0.0046; SD = 0.0025).

Although there are distinct ancient haplotypes that are not recovered among the modern samples, the historical walrus haplotypes reported from Håøya and Bjørnøya appear to be nested among modern haplotypes from the Svalbard archipelago. Furthermore, higher nucleotide sequence variation was recovered from modern eastern Atlantic and Svalbard walruses as compared to the historical samples analyzed here. This might indicate that the intense hunting of the Atlantic walrus at haul-out sites on Bjørnøya and Håøya did not cause an extensive loss of mitochondrial sequence variation and possibly distinct mitochondrial haplogroups (evolutionary closely related haplotypes). However, the current study includes limited sampling and the mitochondrial sequence information provides poor phylogenetic resolution. Hence, it is possible that the relatively lower genetic variation recovered among our historical Atlantic walrus samples may be caused by limited sampling of ancient walrus genetic diversity. For example, the more remote eastern sectors of the Barents Sea may have served as a refugium for East Atlantic walruses during the height of walrus hunting, preserving most of the mitochondrial diversity found within the population today.

## Additional files

10.1186/s13104-016-1907-8 Aligned nucleotide sequences of the mitochondrial cytochrome c oxidase 1 (COI) obtained from historical walrus samples. Reference sequences from other Atlantic walrus samples obtained from GenBank are included.
10.1186/s13104-016-1907-8 Aligned nucleotide sequences of the mitochondrial NADH dehydrogenase 1 (ND1) obtained from historical walrus samples. Reference sequences from other Atlantic walrus samples obtained from GenBank are included.
10.1186/s13104-016-1907-8 Aligned nucleotide sequences of the mitochondrial control region (CR) obtained from historical walrus samples. Reference sequences from other Atlantic walrus samples obtained from GenBank are included.
10.1186/s13104-016-1907-8 List of walrus mitochondrial control region (CR) sequences downloaded from GenBank.
10.1186/s13104-016-1907-8 Maximum likelihood tree obtained from the analyses of the mitochondrial control region haplotypes from historical samples from Bjørnøya and Håøya and modern walrus samples (ATL + LAP + PAC) used in this study. The tree was reconstructed using the RAxML Blackbox tool [30] as implemented on the Cipres Web Portal (http://www.phylo.org). Bootstrap (BS) percentages of ≥50 are indicated.
10.1186/s13104-016-1907-8 Maximum likelihood tree obtained from the analyses of the mitochondrial control region sequences from historical samples from Bjørnøya and Håøya and modern walrus samples (ATL + LAP + PAC) used in this study. The dataset was trimmed in order to minimize the impact of missing information on the analysis. The tree was reconstructed using the RAxML Blackbox tool [30] as implemented on the Cipres Web Portal (www.phylo.org). Bootstrap (BS) percentages of ≥50 are indicated.
10.1186/s13104-016-1907-8 Neighbor network based on all mitochondrial control region sequences from historical samples from Bjørnøya and Håøya and modern walrus samples (ATL + LAP + PAC) used in this study. Nodes are represented by circles, and their sizes are proportional to the number of taxa sharing the haplotype.

## Abbreviations

BIC: Bayesian information criterion
COI: cytochrome c oxidase 1
CR: control region
ESS: effective sample size
MCC: maximum clade credibility
ML: maximum likelihood
ND1: NADH dehydrogenase 1
PCR: polymerase chain reaction
