# Supplementary figures and images for: Genetic diversity of historical Atlantic walruses (Odobenus rosmarus rosmarus) from Bjørnøya and Håøya (Tusenøyane), Svalbard, Norway
Source: BMC Res Notes. 2016 Feb 18;9:112. doi: 10.1186/s13104-016-1907-8 (PMC4757977; doi:10.1186/s13104-016-1907-8)

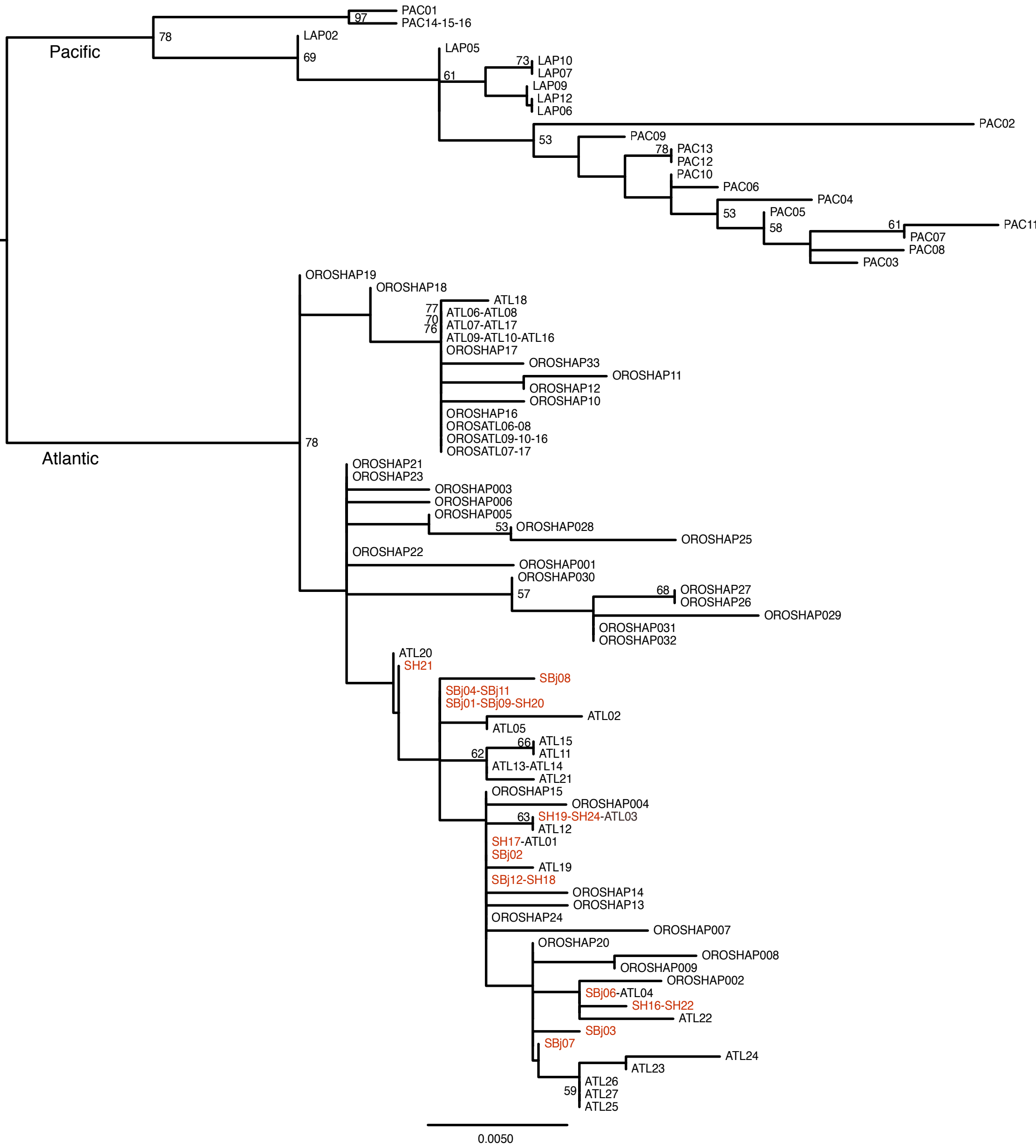

Supplement: Supplementary file 5 — 10.1186/s13104-016-1907-8 Maximum likelihood tree obtained from the analyses of the mitochondrial control region haplotypes from historical samples from Bjørnøya and Håøya and modern walrus samples (ATL + LAP + PAC) used in this study. The tree was reconstructed using the RAxML Blackbox tool [30] as implemented on the Cipres Web Portal (http://www.phylo.org). Bootstrap (BS) percentages of ≥50 are indicated. [file 13104_2016_1907_MOESM5_ESM.pdf]

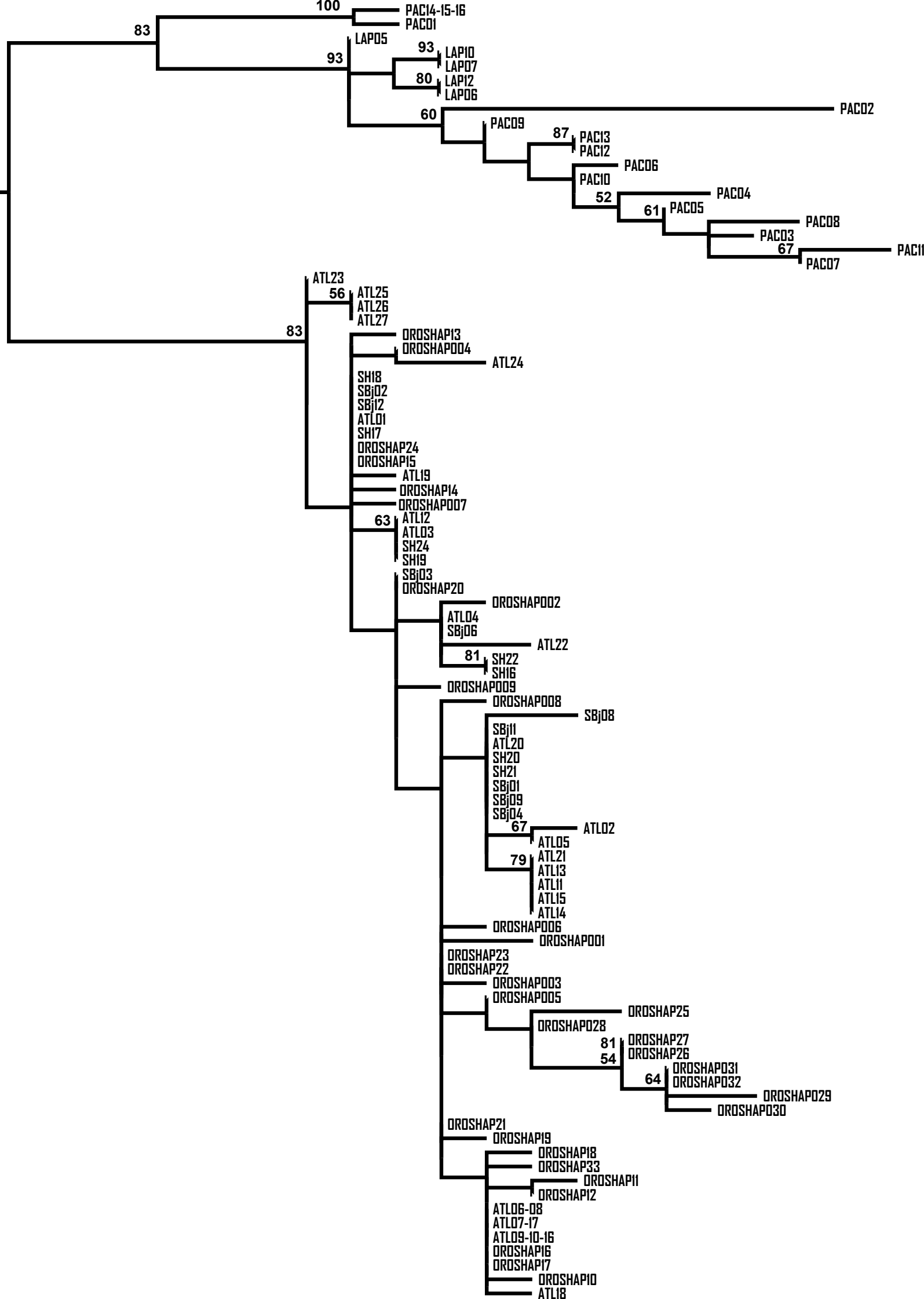

Supplement: Supplementary file 6 — 10.1186/s13104-016-1907-8 Maximum likelihood tree obtained from the analyses of the mitochondrial control region sequences from historical samples from Bjørnøya and Håøya and modern walrus samples (ATL + LAP + PAC) used in this study. The dataset was trimmed in order to minimize the impact of missing information on the analysis. The tree was reconstructed using the RAxML Blackbox tool [30] as implemented on the Cipres Web Portal (www.phylo.org). Bootstrap (BS) percentages of ≥50 are indicated. [file 13104_2016_1907_MOESM6_ESM.pdf]

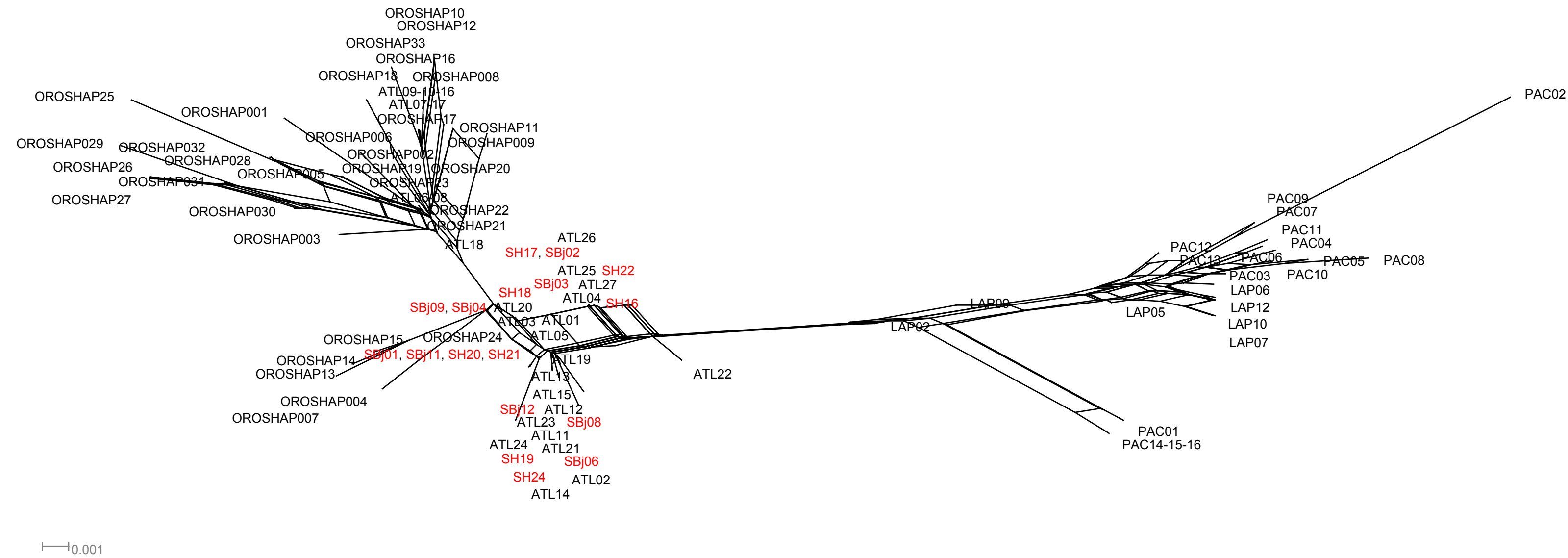

Supplement: Supplementary file 7 — 10.1186/s13104-016-1907-8 Neighbor network based on all mitochondrial control region sequences from historical samples from Bjørnøya and Håøya and modern walrus samples (ATL + LAP + PAC) used in this study. Nodes are represented by circles, and their sizes are proportional to the number of taxa sharing the haplotype. [file 13104_2016_1907_MOESM7_ESM.pdf]
